# Effect of Allelochemicals from Leaf Leachates of *Gmelina arborea* on Inhibition of Some Essential Seed Germination Enzymes in Green Gram, Red Gram, Black Gram, and Chickpea

**DOI:** 10.1155/2014/108682

**Published:** 2014-09-01

**Authors:** Ramakrishnan Madhan Shankar, Shanmugham Veeralakshmi, Abdul Razack Sirajunnisa, Ramasamy Rajendran

**Affiliations:** ^1^Department of Biotechnology, PSG College of Arts and Science, Coimbatore, Tamil Nadu 641014, India; ^2^Department of Chemical Engineering, Annamalai University, Annamalai Nagar, Tamil Nadu 608002, India; ^3^Department of Microbiology, PSG College of Arts and Science, Coimbatore, Tamil Nadu 641014, India

## Abstract

The present work focused on identification of allelochemicals from the leaf leachates of* Gmelina arborea* and analyzing its influence on the germination of red gram, green gram, black gram, and chickpea in terms of the levels of some important germination enzymes like acid phosphatase, catalase, peroxidase, and amylase. The study showed that allelopathic effects were more predominant in chickpea with 80% followed by red gram, green gram, and black gram where the inhibition ranged between 26% and 75%. The vigor index in the seed lots is also considerably reduced. Total chlorophyll content was also reduced by allelopathic effect in all treated seeds ranging between 0.7 and 7.5 *μ*g/g dry weight. The effect of allelochemicals drastically reduced the relative water content in red gram followed by the other seed lots. The total protein content varied considerably in the control and the treated seed lots. Allelochemicals inhibited the expression and activity of the enzymes required for efficient germination. The present study also threw limelight on the effective use of this tree, wherein planting this tree amidst pulse related herb plantations could affect the growth of the economically viable plants, but this tree can very well adapt to diversified soil conditions and rainfall zones.

## 1. Introduction

Allelopathy is the negative effect of chemicals released by one plant species on the growth and reproduction of another [1]. Allelopathy can affect many aspects of plant ecology, including occurrence, growth, and plant succession, the structure of plant communities, dominance, diversity, and plant productivity. These effects can be either positive or negative. There are four main classes of chemical interactions, antibiotics (microorganisms to microorganism), kolines (plants to plants), marasmins (microorganisms to plants), and phytoncides (plants to microorganisms). In allelopathic terms, a chemical is “conveyed” to a “receiver” which can either be “impaired” or “assisted” [2]. Interaction between soil organisms and plants is important in allelopathy. The activity of nitrogen fixing bacteria could be affected by allelopathy. Allelochemicals representing numerous chemical groups have been isolated from over 30 families of terrestrial and aquatic plants. Some Indian plants possessing allelochemicals are* Pongamia glabra* (Karani),* Mathuca indica* (Molarah),* Schleicheria teijuga* (Kusum), and* Celastrus paniculatus* (Malkanguni).

Allelochemicals can cause oxidative stress in the target plants. Therefore, allelochemicals activate the antioxidant mechanism. Autotoxicity occurs when plant species releases toxic chemicals that inhibit germination and growth of same plant species [3]. Allelopathy has also been observed in several tree species.* Leucaena leucocephala*, the tree promoted for revegetation of soil, contains a toxic nonprotein amino acid in the leaves which inhibits growth of others but not its own seedlings. Mimosine is the allelochemical that is released from this tree [4].

*Gmelina arborea* belongs to the family Verbenaceae. It is commonly called Gmelina and white beech (English), Melinaphocal (Spanish), Gamar in Bangladesh, Melina gambar in India, Gmelina in Indonesia, Yemane in Philippines, and Soh in Thailand [5]. It has an extensive geographical distribution in the Indian continent.* Gmelina arborea* is a medium-sized deciduous tree, up to 40 m in length and 140 cm in diameter, but it is usually smaller than other trees. It can show in a wide range of diameter zones ranging from 50 m to 4500 m MSL and in regions of annual rain fall ranging from 50 mm to 9500 mm [6]. Shankar et al. [7] analyzed allelochemicals effect of* Gmelina arborea* on* Vigna mungo* and* Vigna radiata*. The presence of allelopathic compounds such as polyphenols and terpenoids was analysed by TLC and GC-MS. The extract inhibited the proteolytic enzyme important for seed germination. The extract inhibited the germination, seedling growth, and total protein content of both test crops.

The present study focused on identification of the allelochemicals by HPTLC, determination of the effects of the allelochemicals on the germination of black gram, green gram, red gram, and chickpea, and analysis of allelopathic effects by conventional pot experiments. The study also elaborated on determination of the activity of peroxidase, acid phosphatase, amylase, and catalase on various stages of germination and correlating the loss of activity to the detrimental effects of the allelochemicals present in the leaf leachates of* Gmelina arborea*.

## 2. Materials and Methods

### 2.1. Source and Preparation of Leaf Leachate

Fresh leaves and leaf litters of* Gmelina arborea* were collected from a six-year-old tree and stored in polyethene bags in a moisture-free atmosphere. The seeds and plant were identified from the Institute of Forest Genetics and Tree breeding, Coimbatore, Tamil Nadu, India. The leaves were dried in partial shade and stored for the study. A quantity of 50 g of fresh leaf litter was soaked in 159 mL distilled water for 24 hours at 25°C. The leachate was collected and stored at 4°C for further use. Harrington suggested that agricultural seeds retain their longevity in terms of germination efficiency if stored between 0 and 50°C; bringing down the temperature by every 5 degrees, the life of the seed doubles. In the present study, since germination index assessment is the hallmark, retention of inherent viability is essential. Further, orthodox seeds would require lower temperatures and storage at subzero temperatures [8].

### 2.2. Source of Seed for Bioassays

*Vigna mungo* (Green gram Co 7),* Vigna radiata* (Black gram Co 5),* Cajanas cajan* (Red gram Co 6), and* Cicer arietinium* (Chickpea Co 4) required for the seed bioassay were procured from Department of Seed Science and Technology, Tamil Nadu Agriculture University, Coimbatore, Tamil Nadu, India. The seeds were stored in airtight containers, away from moisture at 4°C.

### 2.3. Phytochemical Analysis for Phenolics

Ferric chloride test: 2 mL of water was added to 1 mL of each extract. Two to three drops of 10% ferric chloride solution were added and development of green color was to be observed. Liebermann's test: 1 mL of 20% sulphuric acid was added to 1 mL of each extract followed by addition of few drops of 1% sodium nitrate solution and the tubes were observed for the formation of red color, which on dilution and in alkaline condition with sodium hydroxide turn blue.

### 2.4. HPTLC Analysis of Leaf Leachate

The aqueous extract of leaf sample was centrifuged and the supernatant was collected, which was used as test solution for HPTLC analysis. A volume of 2 *μ*L of the test solution and 3 *μ*L of standard solution was loaded as 6 mm band length in the 3 × 10 silica gel 60F_254_TLC plate using Hamilton syringe and CAMAG LINOMAT 5 instrument. The samples' loaded plate was placed in TLC twin trough developing chamber (after being saturated with solvent vapor) and the plate was developed in the respective mobile phase (toluene-acetone-formic acid (4.5 : 4.5 : 1)) up to 90 mm. The developed plate was dried using hot air to evaporate solvents from the plate. The plate was, then, kept in photodocumentation chamber (CAMAG REPROSTAR 3) to capture the images under white light, UV 254 nm and UV 366 nm. The developed plate was sprayed with a spray reagent (fast blue B reagent) and dried at 120°C in hot air oven. The plate was photodocumented under day light and UV 366 nm using the same photodocumentation chamber. Before derivatization, the plate was fixed in scanner stage and scanning was done at 254 nm. The peak table, peak display, and peak densitogram were noted.

### 2.5. Seed Bioassays

A quantity of 50 g of soil sample was taken in pots of depth of 3 inches and five seeds were sown in each pot. Triplicates were maintained; one seed was taken from each part of the replicate and ground in 3 mL of 0.2 M phosphate buffer (pH 7.0) and centrifuged at 5000 rpm for 10 minutes; the supernatant was collected and used for biochemical analysis. Similarly, four replicates containing five seeds in each pot were maintained to calculate the total chlorophyll content, percentage of germination, germination index, vigour index, root length, and shoot length. The number of seeds germinating was counted everyday up to the sixth day and the percentage of germination was calculated by the formula

(1) $$\begin{array}{c} \text{Germination}\%=\left(\frac{n}{t}\right)*100, \end{array}$$

where *n* is the number of seeds germinated and *t* is the total number of seeds. The germination index was calculated according to Wiese and Binning [9] using the formula

(2) $$\begin{array}{c} \text{GI}=\frac{n}{d}, \end{array}$$

where *n* is the number of seedlings emerging on day “*d*” and *d* is the day after planting. The seed vigour index was calculated by multiplying germination percentage and seedling length (cm). The root length, shoot length, and seedling length were measured using a grading scale on sixth day after germination. The whole plant was weighed after germination, which constituted the fresh weight. The whole plant was wrapped in aluminium foil and placed inside an oven for 48 h at 45°C. The dry weight was measured. The relative water content was calculated at sixth day after germination by

(3) Relative  water  content  (%)  =Fresh  weight−Dry  weightFresh  weight.

The total protein content present in control seeds and treated seed lots was estimated by Bradford's Method (1976) [10].

### 2.6. Total Chlorophyll Content

A quantity of 0.5 g fresh weight of green tissue from both leaf and stem was weighed. The tissue was ground in 10 mL of 95% ethanol. The contents were centrifuged at 5000 rpm for 10 minutes and the supernatant was collected. The absorbance was read at both 647 nm and 664 nm against reagent blank. Total chlorophyll content was checked by Einhelling method [11], using the formulae

(4) Chlorophyll  a  =13.19(ABS  at  664 nm)     −2.57(ABS  at  647 nm) μg/g  dry  weight,Chlorophyll  b    =22.10(ABS  at  647 nm)   −5.26(ABS  at  664 nm) μg/g  dry  weight,Total  chlorophyll  =7.93(ABS  at  664 nm)   +19.53(ABS  at  647 nm) μg/g  dry  weight.

### 2.7. Enzyme Assays

#### 2.7.1. Catalase Assay

The catalase assay was carried out using the method of Chance and Maehly [12]. A volume of 1.9 mL distilled water, 1 mL of 0.059 M hydrogen peroxide, and 0.1 mL of diluted seed extract was added. The decrease in absorbance was recorded at 240 nm for 2-3 minutes and calculated at 240 nm/min from the initial (45 seconds) linear portion of the curve. The enzyme activity was further calculated using the following relationship:

(5) Units/mg =240/min⁡∗100043.6∗mg  enzyme/mL  reaction  mixture.

#### 2.7.2. Determination of Acid Phosphatase

Estimation of acid phosphatase was done according to Malik and Singh [13]. The reaction mixture consisted of 0.5 mL of substrate solution (50 mg of p-nitrophenyl phosphate was dissolved in 10 mL distilled water). The pH was adjusted to 4.8 with 0.5 N NaOH and 0.1 mL of suitably diluted seed extract. The mixture was incubated at 35°C for 30 min. The reaction was stopped by adding 2.4 mL of 0.1 N NaOH. A volume of 0.2 to 1 mL (4 to 20 mM) of the standard (69.75 mg of p-nitrophenol dissolved in 5 mL distilled water to give a final concentration of 100 mM) was diluted to 3 mL with 0.1 N NaOH. The absorbance was recorded at 410 nm against reagent blank. Specific activity was expressed as moles of p-nitrophenol were released per minute per mg of protein.

#### 2.7.3. Estimation of Amylase

Amylase in the sample was estimated according to the technique of  Bernfeld [14]. A volume of 1 mL of 1% starch solution was taken and 1 mL of properly diluted seed extract was added to it. 1 mg/mL of maltose was used as a standard. The contents were incubated at 27°C for 15 minutes. The reaction was stopped by the addition of 2 mL of dinitrosalicylic acid reagent (1 g of dinitrosalicylic acid, 200 mg of crystalline phenol, and 50 mg sodium sulphite in 100 mL 1% NaOH). The solution was heated in a boiling water bath for 5 minutes. A volume of 1 mL of 40% potassium sodium tartrate was added and cooled under running tap water. The absorbance was read at 560 nm against reagent blank. One unit of amylase was expressed as mg of maltose released during 5-minute incubation with 1% starch solution at 27°C.

#### 2.7.4. Peroxidase Assay

Peroxidase was checked using Shannon et al. method [15]. The assay mixture containing 2.5 mL of phosphate buffer, 0.2 mL of suitably diluted seed extract, and 0.1 mL of o-dianisidine (50 mg of o-dianisidine was dissolved in 50 mL of methanol) was incubated at 28°C in a water bath for 2 minutes. The reaction commenced by adding 0.2 mL of H_2_O_2_ (0.6%) and left for 5 minutes. The absorbance was recorded at 430 nm against reagent blank.

## 3. Results and Discussion

**Figure 1:**
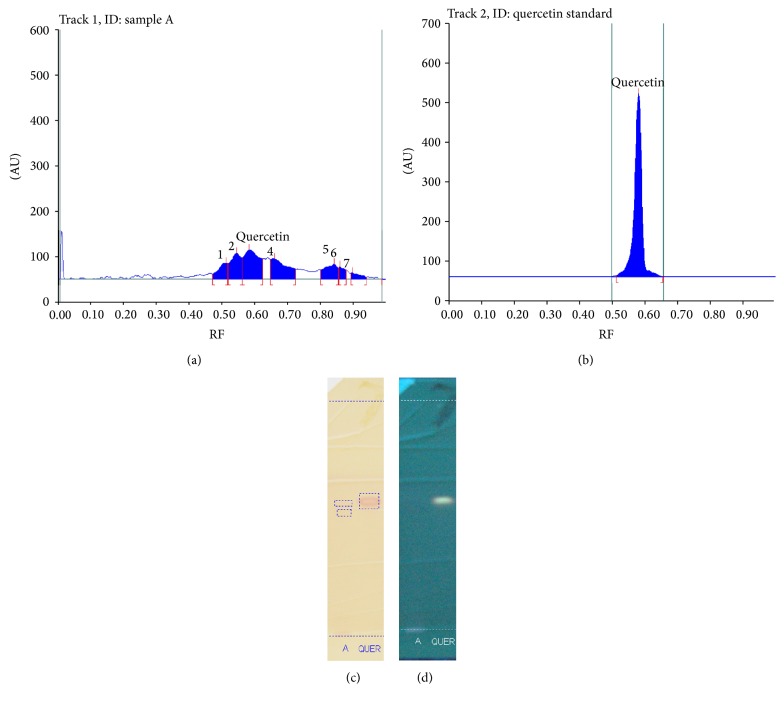
Showing densitograms of leaf leachate (a) and reference Quercetin (b) and chromatograms of HPTLC analysis after derivatization under UV (c) and day light (d).

The leaf leachate of* Gmelina arborea* was subjected to phytochemical analysis that showed a strong positive result for the presence of phenols followed by moderate indication for alkaloids. The aqueous leaf leachate was also subjected to HPTLC analysis and results are shown in Figure 1. Orange brown colored zone at day light was observed after derivatization which confirmed the presence of phenolics having an RF value of 0.51. The standard quercetin was used to compare the unknown sample. Quercetin had an RF value of 0.58. Hence taking into account the closeness of RF values, it could be inferred for the presence of phenolic compounds.

Further, the FTIR spectrum ascertained the presence of phenolic compounds that revealed several peaks ranging between 47.50 cm^−1^ and 2305.01 cm^−1^. This wide range of peaks could be attributed to the presence of various phytochemical constituents in the aqueous leachate representing several bond stretches. Phenols showed the characteristic C=C stretches and C–H vibration for aromatic residues. The strong OH stress band might swamp the weaker C–H stretches band just above 3000 cm^−1^. It is difficult to distinguish a phenol from an aryl alcohol from infrared evidence. Our earlier studies had shown similar results where various phenolic compounds like 3,4,5-hydroxybenzoic acid, 3-(4-hydroxyphenyl) prop-2-enoic acid, and 4-hydroxy-3-methoxybenzoic acid were found to be present in the aqueous leaf leachate [7]. Similarly, several benzoic acid derivatives had been shown to possess potential allelopathic effect on red gram [16].

The crude aqueous leaf leachates were tested for their allelopathic effects on black gram, red gram, chickpea, and green gram. The allelopathic effects were more predominantly seen in chickpea with 80% inhibition of germination (Table 1). In the other systems, namely, black gram, red gram, and green gram, the percentage of inhibition ranged between 26% and 75%. The results were also consistent with earlier report where allelochemicals from Eucalyptus showed around 75% inhibition in red gram [16]. There was also profuse inhibition in terms of reduction in the seedling length. Though only a few seeds in the chickpea seed lot germinated, the sustenance of the plantlet was not very stable as the seedling was watered with the leachate even after germination. Hence, it could be inferred that the allelochemicals of* Gmelina arborea* were not only of inhibitory type to germination but also with retarded plantlet growth after germination. Terzi, 2008, [17] had also observed such kind of high negative inhibition where juglone on allelochemicals from walnut leaf juice significantly retarded the seedling growth of musk melon and cucumber. In the present study, the vigour index observed in various seed lots ranged between 33.2 and 1522 with a mean of 88.3 for black gram, between 124.3 and 1236 with a mean of 687 for red gram, and between 533.8 and 992 with a mean of 785 for green gram seed lots. Since the percentage of inhibition was too high for chickpea seed lot, decipherable vigour index was not observed (Table 1). The results indicated that the leachate influenced more vigour loss in chickpea followed by red gram, black gram, and green gram, respectively. Similar profuse vigour loss was also reported by Pawar and Chavan [18] where the allelopathic effects of Eucalyptus, Melia, Moringa, and Parthenium were observed on wheat, rice, millet, and sorghum.

The effect of allelochemicals of the leaf leachate obtained from* Gmelina arborea* also drastically reduced the relative water content in red gram followed by the other seed lots. The relative water content had a mean of 71.5% (Table 2). Similarly, the dry matter production also considerably reduced in red gram seed lots. This parameter was not accessible for chickpea seed lot where the percentage of survival was almost nil.

**Figure 2:**
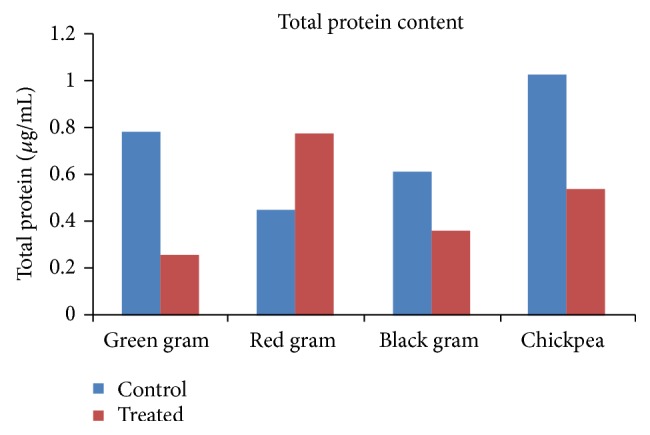
Illustrating the total protein content of the control and samples treated with leaf leachate of* Gmelina arborea*.

The total chlorophyll content also reduced due to allelopathic effect in treated seeds of all the seed lots ranging between 0.7 and 7.5 *μ*g/g dry weight. Similarly, the total protein content also varied between 1.02 and 0.45 in the control and between 0.25 and 0.44 in the allelochemicals treated seed lots (Figure 2).

**Figure 3:**
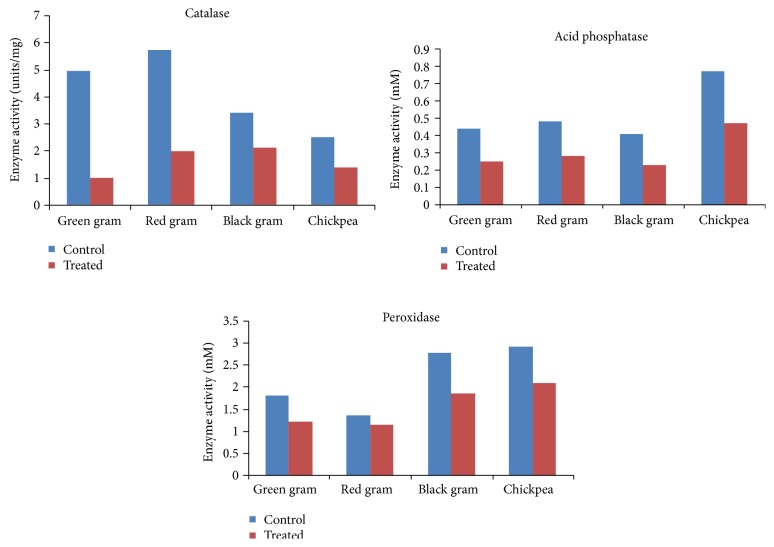
Activity of various enzymes in germination of control and samples treated with leaf leachate of* Gmelina arborea*.

Enzymes are one of the chief molecules, which are stimulated when a seed germinates; hence it would be very appropriate in inhibiting the enzymes like acid phosphatase, catalase, peroxidase, and amylase whose expression is thought to be upregulated during seed germination. In the present study, activity of enzyme was found to increase as days of germination increased; for instance, the level of peroxidase in control red gram was 0.76 units (Figure 3). This trend increased exponentially after the second day of germination reaching a peak of 1.97 units in control, after 6th day of germination.

The inhibition of phosphatase directly affects the energy metabolism and ATP production and contributes to the seed vigour and retardation is visible in germination. Senna et al., 2005, [19] had reported the favorable role of acid phosphatase in maize seeds germination where the activity of the acid phosphatase increased in 24 h of onset of germination. Similarly, the levels of prolyl aminopeptidase and acid phosphatase were higher in germinating seeds of* L. esculenta*. These enzymes have the potential role in liberation of proline and other amino acids that are released by the enzymatic hydrolysis of several seed storage proteins. Matveyeva et al., 2003, [20] had reported on several compounds like furosemide, bumetanide, and so forth, which are inhibitors of calcium cotransporters to suppress the germination rate to less than 50%. There is potential role of EMP enzymes particularly dehydrogenases in seed germination upon treatment with inhibitors.

**Figure 4:**
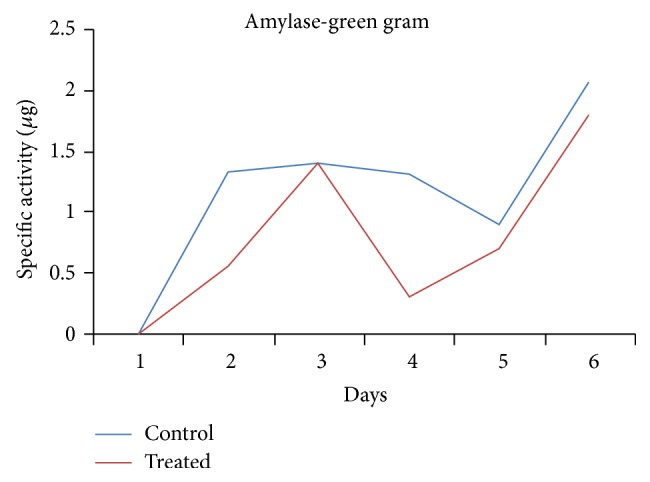
Activity of amylase observed in green gram.

Further, the activity of these enzymes was also not stable after the plant has started the photosynthesis. Similar trends were also observed with the activity of amylase being seen only in green gram (Figure 4). This explains that in every individual type of seed different enzymes either singly or in combination are responsible for an efficient germination and plantlet establishment, all with a common role to play, namely, release of amino acids and other small molecules from the stored proteins and carbohydrates in the seed, which could be used for germination.

## 4. Conclusions

The present study showed that the allelopathic compounds present in the leaf leachate of* Gmelina arborea* such as phenolics and 4-hydroxy-3-benzoic acid inhibited crucial enzymes responsible for seed germination. Compared to the studied four seed lots, the germination was inhibited in chickpea predominantly. The addition of leaf leachate to the germinating seed also affected the seedling length. The relative water content and total chlorophyll content were varied from 81% to 50% and from 97% to 64% in control and treated seed lots. The vigour index represents the viability of seeds. The present study indicated that vigour index also varied and it was much skewed in black gram seed lots. The protein content also got affected. The present study also revealed that the allelochemicals released from* Gmelina arborea* affected the germination and growth of black gram, green gram, red gram, and chickpea. Thus, the extract reduced the growth of economically important seeds. In the future study,* Gmelina arborea* allelochemicals could be used in the control of weeds. The present study also threw limelight on the effective use of this agroforestry tree, wherein planting this tree amidst pulse related herb plantations could affect the growth of the economically viable plants. Potential plantations of* Gmelina arborea* could be tried in uncultivable dry lands as this species can very well adapt to diversified soil and climatic conditions.

## Figures and Tables

**Table 1:** Effect of leaf leachate of *Gmelina arborea* on germination of seeds and seedling growth.

Samples	Seedling length (cm)		Germination percentage (%)		Vigour index	
	Control	Treated	Control	Treated	Control	Treated
Green gram	14.46	10.46	80	75	1157	785.2
Red gram	13.60	10.57	85	65	1156	687.3
Black gram	18.02	12.18	95	72.5	1712	883
Chickpea	9.85	—	60	—	591	—

**Table 2:** Relative water content of leaf leachate treated and untreated seed lots.

Samples	Seedling fresh weight (mg)		Seedling dry weight (mg)		Relative water content (%)	
	Control	Treated	Control	Treated	Control	Treated
Green gram	176.53	58.45	27.84	16.38	84.23	71.98
Red gram	49.07	17.04	18.45	9.33	60.82	45.26
Black gram	266.54	92.17	30.12	18.97	88.70	79.42
